# The natural selection of metabolism and mass selects lifeforms from viruses to multicellular animals

**DOI:** 10.1002/ece3.3432

**Published:** 2017-09-27

**Authors:** Lars Witting

**Affiliations:** ^1^ Greenland Institute of Natural Resources Nuuk Greenland

**Keywords:** allometry, body mass, evolution, life history, major transition, metabolism

## Abstract

I show that the natural selection of metabolism and mass can select for the major life‐history and allometric transitions that define lifeforms from viruses, over prokaryotes and larger unicells, to multicellular animals. The proposed selection is driven by a mass‐specific metabolism that is selected as the pace of the resource handling that generates net energy for self‐replication. An initial selection of mass is given by a dependence of mass‐specific metabolism on mass in replicators that are close to a lower size limit. A sublinear maximum dependence selects for virus‐like replicators, with no intrinsic metabolism, no cell, and practically no mass. A superlinear dependence selects for prokaryote‐like self‐replicating cells, with asexual reproduction and incomplete metabolic pathways. These self‐replicators have selection for increased net energy, and this generates a gradual unfolding of population‐dynamic feed‐back selection from interactive competition. The incomplete feed‐back selects for larger unicells with more developed metabolic pathways, and the completely developed feed‐back for multicellular animals with sexual reproduction. This model unifies the natural selection of lifeforms from viruses to multicellular animals, and it provides a parsimonious explanation where allometries and major life histories evolve from the natural selection of metabolism and mass.

## 1 INTRODUCTION

Following the origin of replicating molecules, the living matter on Earth evolved into large organisms with organized lifeforms. Yet, the natural selection mechanisms that caused the evolution have remained largely unknown.

The actual routes of evolution were dependent on specific event (Maynard Smith & Szathmáry, 1995). The emergence of cells with metabolism allowed simple lifeforms to fuel their own replication. The inclusion of mitochondria and chloroplasts allowed for a more efficient metabolism. The emergence of multicellularity allowed for the development of an organized physiology. And the emergence of sexual reproduction allowed the genome to reorganize into new combinations.

These traits were favored in evolution, not because they emerged by mutation, nor because they were beneficial to the organism per se, but because they were naturally selected. But how could they become naturally selected, when large and multicellular sexual organisms have a slow rate of replication, and natural selection favors fast replication?

This paradox, that living matter arose as replicating molecules with a persistent natural selection for fast replication, and evolved in the opposite direction, calls for a critical rethinking of evolutionary biology. To successfully explain the living world, we need to show that the origin of replicating molecules generates a natural selection that evolves into a more advanced form of selection that continues to select, not only for fast replicators at the molecular level, but also for the large and organized lifeforms that inhabit the Earth today.

The development of such a theory is the aim of this article. To approach the goal, I use the framework of Malthusian relativity (MR, see Witting, 1997, 2008, 2017) to deduce a natural selection that is sufficient to explain the metabolism, body masses, major life‐history transitions, and interspecific allometries that are observed within and across lifeforms from viruses over prokaryotes and larger unicells to multicellular animals. The proposed selection describes how mobile lifeforms with elevated metabolic rates, large body masses, and reduced sexual reproduction are selected as a deterministic consequence of the origin of replicating molecules.

The original version of MR describes how the selection of net energy for self‐replication, and the selection of mass by the intraspecific and density‐dependent interactive competition, explains the evolution of major life‐history transitions (Witting, 1997, 2002a, 2007, 2008) and allometric exponents in mobile animals (Witting, 1995). This selection involves a feed‐back, where the density dependence of interactive competition transforms population‐dynamic growth (as induced by the selected net energy) into a persistent density–frequency‐dependent selection of mass and other life histories (Witting, 2000, 2002b). I refer to this selection as the population‐dynamic feed‐back selection of interactive competition, and it allows large organisms with a slow sexual reproduction to evolve from the intrapopulation selection of fast replication. The original mechanism, however, did not incorporate primary selection on metabolism, and nor did it allow for a selection differentiation between small replicators like virus, prokaryotes, and larger unicells.

The primary selection of metabolism was recently incorporated into the feed‐back selection of MR in relation to the evolution of interspecific allometries (Witting, 2017). This showed that mass‐specific metabolism can be selected as a proxy for the pace of the resource handling that generates net energy for self‐replication. The net energy generates population growth and interactive selection for larger body masses, with the joint selection of metabolism and mass selecting for a range of transitions in the interspecific allometries that are observed in taxa from prokaryotes over larger unicells to multicellular animals.

By describing the physiological and ecological constraints on the selection of metabolism and mass, Witting (2017) was able to solve the resulting equations and predict the range of allometric exponents from first principles of self‐replication. This showed how the optimal density regulation and physiological trade‐offs link selection on the home range and population density to selection on metabolism, life periods, net energy, and mass. But it did not explain the specific selection of metabolism and mass that will make the selection equations unfold into a range of lifeforms with different body masses, allometries, and life histories.

The latter requires a mechanism where the origin of replicating molecules selects for an increase in metabolism and mass, with a succession of transitions in the mechanism of natural selection that selects the transitions in the life‐history and allometric exponents that are observed between virus, prokaryotes, larger unicells, and multicellular animals. It is this evolution that is studied in the this article, where the transitions between the four lifeforms are predicted as a directional evolutionary succession from the primary selection of mass‐specific metabolism and mass.

## 2 BACKGROUND

The majority of MR is developed in earlier work (Witting, 1995, 1997, 2007, 2008), with the this article being an extension of Witting (2002a, 2017). A complete understanding of the proposed selection requires familiarity with the latter two papers that deal with the natural selection of major life‐history (Witting, 2002a) and allometric (Witting, 2017) transitions.

This background section summarizes the earlier work, providing a conceptual framework for an easier understanding of the details that follow in the sections below. Model parameters and basic relations are defined in Table 1, with the range of selection attractors listed in Table 3.

**Table 1:** Important symbols (S) with SI units and basic relationships, including the interpretation of scripts and accents

S	SI	Basic relations	Description
*w*	J	$\frac{\partial \ln w}{\partial \ln\epsilon }=1/\hat{\epsilon }$	Body mass of individual in joule (combustion energy).
ln *w*	‐	lnw=ln[w/(1J)]	Natural logarithm of mass.
β	J/Js	$\beta \propto \beta_{\beta }\beta_{w}$	Mass‐specific metabolism; $\beta_{\beta }$: primary selected; $\beta_{w}$: mass‐rescaling selected.
$\tilde{\beta }$	1/s	$\tilde{\beta }=\beta /W$	Metabolic pace in physical time.
*W*	J/J	*W* = 1J/J	Mass‐specific work of one joule metabolized per unit mass.
*x*	‐	$x=x_{0}w^{\hat{x}},\;\hat{x}={\hat{x}}_{\beta }+{\hat{x}}_{w}$	Interspecific allometry for trait x; $x_{0}$: intercept; $\hat{x}$: exponent.
$x_{\beta }$	‐	$x_{\beta }=w^{{\hat{x}}_{\beta }}$	Metabolic‐rescaling allometry (interspecific).
$x_{w}$	‐	$x_{w}=w^{{\hat{x}}_{w}}$	Mass‐rescaling allometry (interspecific).
*t*	s		Physical time.
τ	G	$\tau =t/t_{g}$	Biotic time, in generations (G).
$t_{x}$	s	$t_{x}=\tau_{x}t_{g},\;x\;:\;l,g,m,j,r$	l: lifespan, g: generation, m: maturity, j: juvenile & r: reproductive period.
$\tau_{x}$	G	$\tau_{x}=t_{x}/t_{g},\;x\;:\;l,g,m,j,r$	l: lifespan, g: generation, m: maturity, j: juvenile & r: reproductive period.
ρ	J/m${}^{d}$	$\rho =f\rho_{u}$	Realized resource per unit d dimensional habitat. $\rho_{u}$: unexploited resource.
f	‐	$f=f_{e}f_{\iota }f_{s}$	Density regulation by exploitation ($f_{e}$), interference ($f_{\iota }$), & self‐inhibition ($f_{s}$).
α	J	$\alpha =\overset{`}{\alpha }\rho^{**}$	Handling of net resource assimilation. $\overset{`}{\alpha }$: intrinsic handling (Jm^*d*^/J).
$\tilde{\alpha }$	1/s	${\tilde{\alpha }}^{**}=\tilde{\beta }$	Pace of resource handling; selected to resemble metabolic pace.
ϵ	J/s	$\epsilon =\alpha \tilde{\alpha }=\alpha \tilde{\beta }$	Net assimilated energy (energetic state) per individual per unit *t* time.
$\epsilon_{g}$	J/s	$\epsilon_{g}=\epsilon +\beta w$	Gross assimilated energy per individual per unit *t* time.
$r_{x}$	1/G	$r_{x}=\frac{d\ln x}{d\tau },\;x\;:\;\alpha ,\beta_{\beta },\epsilon$	Per‐generation exponential increase in α, $\beta_{\beta }$ & ϵ. $r_{\epsilon }=r_{\alpha }+r_{\beta_{\beta }}$.
*p*	‐	$p=R_{0}/R$	Probability to survive to reproduce.
*m*	1/s	$m=\epsilon /\overset{´}{\beta }w$	Reproductive rate in physical time.
*R*	‐	$R=t_{r}m,\;R^{*}=1/p^{*}$	Lifetime reproduction.
*R*_0_	‐	$R_{0}=pR$	Expected lifetime reproduction.
λ	‐	$\lambda =pR,\;\lambda^{*}=1$	Population growth; per‐generation multiplication factor.
*r*	1/G	$r=\ln\lambda =\frac{d\ln n}{d\tau },\;r^{*}=0$	Population growth; per‐generation exponential increase.
$\overset{´}{\beta }$	‐	$\overset{´}{\beta }=1+{\overset{´}{w}}_{j}{\overset{´}{\beta }}_{j}/\overset{´}{\dot{w}}$	Invariant scaling of reproduction to account for offspring metabolism.
$\beta_{j}$	J/Js	${\overset{´}{\beta }}_{j}=\beta_{j}/\beta$	Average mass‐specific metabolism of offspring during the juvenile period $t_{j}$.
$w_{j}$	J	${\overset{´}{w}}_{j}=w_{j}/w$	Average mass of offspring during $t_{j}$.
$\dot{w}$	J/s	$\overset{´}{\dot{w}}=\dot{w}/w\beta$	Average ontogenetic growth during $t_{j}$.
*d*	‐		Spatial habitat dimensions for interactive foraging behavior. 1D, 2D & 3D.
*n*	1/m${}^{d}$		Population density; individuals per unit *d* dimensional habitat.
*I*	1/s		Intraspecific interference; competitive encounters per individual per unit *t* time.
ι	‐	$\iota^{**}=\frac{4d-1}{2d-1}\frac{1}{\psi },\;\iota^{\bar{*}*}=\frac{1}{\psi }$	Log intraspecific interference, ι=lnI. ${\bar{\iota }}^{*}$: mass dependent maximum.
ψ	‐		Fitness cost gradient per unit interference across body mass variants.
*h*	m${}^{d}$		Home range of individual in d habitat dimensions.
*v*	m/s	$v=v_{0}\beta_{\beta }w^{\hat{v}},\;\hat{v}=\hat{t}$	Foraging speed of individual in physical time.
$\sigma_{\ln w}^{2}$	‐		Additive heritable variance of a trait, here w on log scale.

Relation	Script	
Modification	Accent	$\tilde{x}$: pace of x; $\overset{´}{x}$: relative, fractional, or conglomerate x; $\overset{`}{x}$: intrinsic x; $\dot{x}$: dx/dt.
Allometry	Subscript/accent	β: metabolic‐rescaling; w: mass‐rescaling; ∘: intercept; $\hat{x}$: $w^{\hat{x}}$ exponent; $\overset{ˇ}{x}$: $\beta^{\overset{ˇ}{x}}$ exp.
Density regulation	Subscript	e: exploitation; ι: interference; s: local exploitation; u: unregulated; ∘: intercept.
Life periods/ages	Subscript	j: juvenile; m: maturity; r: reproduction; g: generation; l: lifespan.
Attractors	Superscript	∗: population‐dynamic equilibrium; ∗∗: selection attractor.
Constraints	Bar	$\underline{x}$: lower limit on x; $\bar{x}$: upper limit on x.

I will use the same life‐history model to cover the entire span of organisms. It is based on a formulation

(1) $$\lambda^{*}=pR=pt_{r}\epsilon /\overset{´}{\beta }w=1$$

of the per‐generation replication (λ) of an average variant in an age‐structured population at the population‐dynamic equilibrium (denoted by superscript *), where *p* is the probability to survive to reproduce, $R=t_{r}\epsilon /\overset{´}{\beta }w$ is lifetime reproduction (unitless number), *t*_*r*_ the reproductive period in physical time (SI unit s), ε the density‐dependent net energy that is available for self‐replication per unit physical time (SI unit J/s), *w* the body mass as measured by biotic (combustion) energy (SI unit J), and $\overset{´}{\beta }$ a unitless scaling parameter that accounts for energy that is metabolized by the offspring (see Witting, 2017 for details).

Given stable conditions with unconstrained selection, the life history of Equation (1) selects (∂r/∂lnϵ=1, with r=lnλ) for an exponential increase in net energy

(2) $$d\ln\epsilon /d\tau =\sigma_{\ln\epsilon }^{2}\partial r/\partial \ln\epsilon =\sigma_{\ln\epsilon }^{2}$$

on the per‐generation time‐scale (τ) of natural selection, with $\sigma_{\ln\epsilon }^{2}$ being the additive genetic variance. Independently of the selection cause for the evolution of mass, the individuals of an evolutionary lineage cannot be large unless they have evolved the ability to consume plenty of resources. This implies a change in mass that is selected, in one way or the other, as a consequence of the evolutionary change in net energy, that is,

(3) $$\frac{d\ln w}{d\tau }=\frac{\partial \ln w}{\partial \ln\epsilon }\frac{d\ln\epsilon }{d\tau }$$

with

(4) $$\frac{\partial \ln w}{\partial \ln\epsilon }=\frac{1}{\hat{\epsilon }}$$

being the selection dependence of mass on energy given a log‐linear relation, where $\hat{\epsilon }$ is an invariant parameter. Natural selection is thus selecting mass as an evolutionary consequence of net energy

(5) $$w=\int \frac{\partial \ln w}{\partial \ln\epsilon }d\ln\epsilon \propto \epsilon^{1/\hat{\epsilon }},$$

with $\hat{\epsilon }$ being the exponent of the body mass allometry for net energy, where $\epsilon \propto w^{\hat{\epsilon }}$ with accent that denoting the allometric exponent of the underlying trait, here ϵ (Witting, 2017).

To obtain a better understanding of the selection of mass, we need to consider the underlying mechanisms that generate the net energy that is selected into mass. This net energy of self‐replication is a product ($\epsilon =\alpha \tilde{\alpha }$) between an ecological/physiological mechanical/biochemical handling of resource assimilation (α, resource handling in short, SI unit J), and the pace ($\tilde{\alpha }$, SI unit 1/s; with tilde accent denoting pace) of this process. The pace of handling is defined $\tilde{\alpha }={\overset{´}{\epsilon }}_{\alpha }/W$ by the mass‐specific energy (${\overset{´}{\epsilon }}_{\alpha }=\epsilon_{\alpha }/w$, SI unit J/Js; $\epsilon_{\alpha }$: total handling energy; SI unit J/s) that is metabolized for handling per unit time, divided by the mass‐specific work (*W*; SI unit J/J) of one joule metabolized for handling per unit mass. This energy ${\overset{´}{\epsilon }}_{\alpha }=c\beta$ is the fraction (0 ≤ *c* ≤ 1) of the mass‐specific metabolism (β; SI unit J/Js) that is used for the handling of resources.

Net energy is also the difference between gross energy ($\epsilon_{g}$) and the metabolic cost (wβ) of the individual, with $\epsilon =\epsilon_{g}-w\beta =\alpha_{g}c\beta /W-w\beta$. From the positive partial derivatives ∂r/∂lnϵ=1 and $\partial \epsilon /\partial c=\alpha_{g}\beta /W$, there is selection for a pace of handling that is as large as possible, with a selection attractor where c=1, ${\overset{´}{\epsilon }}_{\alpha }=\beta$, and the pace of handling $\tilde{\alpha }=\tilde{\beta }$ is the pace of metabolism, with $\tilde{\beta }=\beta /W$. This implies a mass‐specific metabolism that is selected as the pace of the resource handling that generates net energy for self‐replication, where

(6) $$\epsilon =\alpha_{g}\tilde{\beta }-w\beta =\alpha \tilde{\beta }$$

with $\alpha =\alpha_{g}-wW$.

The exponential increase in net energy

(7) $$\frac{d\ln\epsilon }{d\tau }=\frac{d\ln\alpha }{d\tau }+\frac{d\ln\beta }{d\tau }$$

is thus driven by the selected increase in the two subcomponents of resource handling and mass‐specific metabolism, with the energetic exponent ($\hat{\epsilon }=\hat{\alpha }+\hat{\beta }$) for the selected mass ($w\propto \epsilon^{1/\hat{\epsilon }}$) matching the sum of the exponents for resource handling and mass‐specific metabolism.

To include the underlying mechanisms for the selection of mass and allometric exponents, we need to connect the life history to the underlying resources that supply the organism with energy. This is done by resource handling ($\alpha =\overset{`}{\alpha }\rho^{**}$) that is defined here for an implicit resource (ρ) at the density (n) regulated [$\rho =\rho_{u}f\left(n\right)$; $\rho_{u}$: unexploited resource; f(n): density regulation] population‐dynamic equilibrium of the relevant selection attractor for mass (denoted by superscript **), with $\overset{`}{\alpha }$ being an intrinsic handling component that specifies the energy that is obtained by handling as a function of the resource.

A simple joint density regulation function like f(n), however, is insufficient for the evolutionary deduction of mass and allometries. For this, we need to partition ($f=f_{e}f_{\iota }f_{s}$) regulation into population exploitation ($f_{e}$), regulation by interference competition ($f_{\iota }$), and the local exploitation of the individual ($f_{s}$). This frequency‐independent regulation is then used as a starting point for a description of the density–frequency‐dependent bias that is created by interactive competition in the net assimilated energy across the heritable variants in the population [see e.g., Equation (19)]; a bias that is selecting for large body masses and associated life‐history transitions (Witting, 1997, 2002a).

The exponents of the body mass allometries are selected from the ecological and physiological constraints on this selection of mass (Witting, 1995, 2017). One of the most important constraints in this selection is a density‐dependent foraging that is optimized for a trade‐off between the regulation of interference competition and the local exploitation of the resource, with the resulting allometric exponents (Table 2) being dependent on the spatial dimensionality of the interactive foraging behavior.

Irrespectively of the underlying details of the natural selection of mass, the average mass of a selection attractor can be expressed as

(8) $$w^{**}=pt_{r}\epsilon /\overset{´}{\beta }$$

from a rearrangement of Equation (1), with the dependence of mass on net energy being

(9) $$w^{**}\propto \epsilon^{2d/(2d-1)},$$

where $\hat{\epsilon }=2d/\left(2d-1\right)$ (Table 2), and d∈{1,2,3} is the spatial dimensionality of the interactive behavior.

**Table 2:** Theoretical allometries. Allometric exponents ($\hat{x}$) as they evolve from allometric rescaling given primary selection on metabolism and mass. The exponents depend on the dimensionality of the interactive behavior and on the ${\hat{\beta }}_{\beta }$ exponent that describes the relative importance of mass‐specific metabolism for the net energy of the organism. Symbols: ϵ: net energy; α: resource handling; β: mass‐specific metabolism; t: biotic periods in physical time; *p*: survival; R: lifetime reproduction; r: population growth rate; h: home range; n: population density. From Witting (2017)

${\hat{\beta }}_{\beta }$	$\hat{\alpha }$	$\hat{\beta }$	$\hat{t}$	$\hat{p}$	$\hat{R}$	$\hat{r}$	$\hat{h}$	$\hat{n}$
(a) One‐dimensional interactions ($\hat{\epsilon }=1/2$)								
0	1	$-\frac{1}{2}$	$\frac{1}{2}$	0	0	$-\frac{1}{2}$	1	$-\frac{1}{2}$
$\frac{1}{2}$	$\frac{1}{2}$	0	0	$\frac{1}{2}$	$-\frac{1}{2}$	0	1	−1
1	0	$\frac{1}{2}$	$-\frac{1}{2}$	1	−1	$\frac{1}{2}$	1	$-\frac{3}{2}$
(b) Two‐dimensional interactions ($\hat{\epsilon }=3/4$)								
0	1	$-\frac{1}{4}$	$\frac{1}{4}$	0	0	$-\frac{1}{4}$	1	$-\frac{3}{4}$
$\frac{1}{4}$	$\frac{3}{4}$	0	0	$\frac{1}{4}$	$-\frac{1}{4}$	0	1	−1
$\frac{1}{2}$	$\frac{1}{2}$	$\frac{1}{4}$	$-\frac{1}{4}$	$\frac{1}{2}$	$-\frac{1}{2}$	$\frac{1}{4}$	1	$-\frac{5}{4}$
1	0	$\frac{3}{4}$	$-\frac{3}{4}$	1	−1	$\frac{3}{4}$	1	$-\frac{7}{4}$
(c) Three‐dimensional interactions ($\hat{\epsilon }=5/6$)								
0	1	$-\frac{1}{6}$	$\frac{1}{6}$	0	0	$-\frac{1}{6}$	1	$-\frac{5}{6}$
$\frac{1}{6}$	$\frac{5}{6}$	0	0	$\frac{1}{6}$	$-\frac{1}{6}$	0	1	−1
$\frac{1}{2}$	$\frac{1}{2}$	$\frac{1}{3}$	$-\frac{1}{3}$	$\frac{1}{2}$	$-\frac{1}{2}$	$\frac{1}{3}$	1	$-\frac{4}{3}$
1	0	$\frac{5}{6}$	$-\frac{5}{6}$	1	−1	$\frac{5}{6}$	1	$-\frac{11}{6}$

The allometric exponent for net energy ($\hat{\epsilon }$) is special in the sense that it does not depend on the underlying causes (α versus β) for the net energy that is selected into mass (Witting, 2017). The allometric exponents

(10) $$\hat{x}={\hat{x}}_{\beta }+{\hat{x}}_{w}$$

for most traits (*x*: unspecified trait), however, are dependent on the relative importance of metabolism for the selection of mass, with the final exponent ($\hat{x}$) being the sum of two subexponents that evolve from the metabolic‐rescaling (${\hat{x}}_{\beta }$) and mass‐rescaling (${\hat{x}}_{w}$) selection of the life‐history (Witting, 2017). The former is denoted by subscript β, and it is given by the primary selection of mass‐specific metabolism, the associated dependence of rate‐dependent life‐history traits on the selected changes in metabolic pace, and the dependence of the selected mass on the net energy that is generated by the primary selection of metabolism.

The second process of mass‐rescaling (denoted by subscript w) is the direct selection response of the life‐history to the evolutionary changes in mass. It is initiated by a metabolic trade‐off that selects for a decline in metabolism and a dilation of the reproductive period with an increase in mass, in order to prevent a decline in net energy and fitness on the per‐generation time‐scale of natural selection. This mass‐rescaling selects the well know Kleiber (1932) allometries as the life‐history response when there is no primary selection on mass‐specific metabolism (i.e., when ${\hat{\beta }}_{\beta }=0$), with 1/4 exponents being the two‐dimensional case of the more general 1/2*d*. The final exponents ($\hat{x}$) are then selected to change with an increase in the importance of mass‐specific metabolism for the selection of mass, as described by a ${\hat{\beta }}_{\beta }$ exponent that increases from zero to one (see Table 2).

The theory behind these equations makes it possible to have a single model that will predict diverse lifeforms from the primary selection on metabolism, net energy, and mass. The most basic selection for this is maybe the mass‐specific metabolism that is selected as the pace of the resource handling that generates net energy for self‐replication (Witting, 2017). The selection of mass is then following from the increase in net energy, either by a dependence of metabolism on mass in self‐replicators that are close to a lower size limit, and/or by the density‐dependent interactive competition that is generated by the population growth that follows from the average net energy in the population. The associated transitions in the interactive selection of mass is selecting for major life‐history transitions (see Witting, 2002a for details), and body mass allometries are selected as a response to the primary changes in metabolism and mass (Witting, 2017). It is this joint selection that is studied in details below, with most of the evolutionary transitions between lifeforms following from a gradual development of the population‐dynamic feed‐back selection of the density‐dependent interactive competition.

## 3 REPLICATING MOLECULES

My starting point for evolution is the origin of replicating molecules with no intrinsic metabolism (β=0), no cell, and practically no mass (*w*), with underline denoting the lower limit. Replication at the origin is driven by an extrinsic source of energy (metabolism), with the rate of self‐replication (Equation (1)) being zero as $\epsilon =\alpha \tilde{\beta }=0$. While the replication of these molecules requires energy, they are essentially zero‐energy replicators as they have no intrinsic metabolism to generate energy.

The molecular replicator may evolve into a self‐replicating cell with an internal metabolism that generates net energy for self‐replication, with the β=0↔β>0 transition separating life histories with no energy (ϵ=0) from those with energetic states (ϵ>0).

### 3.1 Initial metabolism depends on mass

The initial selection of metabolism and mass may occur by a dependence of mass‐specific metabolism on mass when mass is close to an absolute minimum (DeLong, Okie, Moses, Sibly, & Brown, 2010). This is because the metabolism that generates the net energy that is required for self‐replication is dependent at least upon the mass of the involved metabolic molecules.

Extremely low levels of metabolism are not necessarily dependent upon the development of a compartment like a cell. But it is assumed here that a self‐replicator with an advanced form of metabolism is dependent upon a cell‐like structure where the different molecules of the metabolic pathways can concentrate (e.g., Koch & Silver, 2005; Maynard Smith & Szathmáry, 1995; Michod, 1999; Miller & Orgel, 1974; Oparin, 1957; Wächtershäuser, 2000). Then, with selection driven by the self‐replication energy that is generated by metabolism, it follows that the evolution of the self‐replicating cell is the evolution of a metabolic compartment. The increase in net energy for self‐replication with increased mass‐specific metabolism can then be the selection that drives the evolution of the cell and all its associated mass; let it be the mass of the cell membrane, over the heritable code, to the metabolic molecules themselves.

The initial evolution of mass‐specific metabolism is thus linked to the evolution of the cell and its mass, simply because the metabolism cannot evolve without the mass. This mass dependence of mass‐specific metabolism is not expected to vanish immediately with the evolution of a self‐replicating cell, because larger cells allow for the evolution of more fully developed metabolic pathways. The mass dependence, however, will eventually vanish with the evolution of complete metabolic pathways in larger cells.

To describe this in more detail let us assume that the smallest self‐replicators have a passive interactive competition that cannot generate anything but an insignificant bias in the distribution of resources over mass, given the level of interference in the population. Body mass selection is then frequency‐independent, and the partial selection gradient

(11) $$\partial r/\partial \ln w_{|\beta }=-1$$

across self‐replicating variants with a given mass‐specific metabolism (subscript |β) is negative, because of the quality–quantity trade‐off ($R\propto 1/\overset{´}{\beta }w$) of Equation (1). For this selection, let ${\underline{w}}_{\beta }^{*}$ be the partial selection attractor of the minimum mass ($\underline{w}$) that is required to sustain a given mass‐specific metabolism β.

A low‐energy self‐replicator that is evolving a cell with given metabolic pathways may thus be considered to evolve along a gradient of β‐dependent minimum masses (${\underline{w}}_{\beta }^{*}$), with each mass being selected by Equation (11) as the minimum mass that is required to sustain a self‐replicator with the given mass‐specific metabolism. Smaller masses cannot sustain the metabolism, and larger masses with the same mass‐specific metabolism are selected toward the gradient of β‐dependent minimum masses because of the quality–quantity trade‐off.

The selected mass‐specific metabolism is then an increasing function of the β‐dependent minimum mass, that is,

(12) $$\partial \ln\beta /\partial \ln{\underline{w}}_{\beta }^{*}=\hat{\beta }$$

where $\hat{\beta }$ is a positive exponent that declines as the increase in the β‐dependent minimum mass provides more and more fully developed metabolic pathways.

An initial selection increase in net energy by an increase in metabolic pace will thus be inherently linked to an increase in the β‐dependent minimum mass. But when is the positive dependence of mass‐specific metabolism on minimum mass strong enough to generate the extra net energy that is needed, not only for the production of larger offspring, but also for the production of more offspring; as required before a selection increase in metabolism and mass can occur?

### 3.2 The initial selection

To examine this, we have $\hat{\beta }={\hat{\beta }}_{\beta }+{\hat{\beta }}_{w}$, where ${\hat{\beta }}_{\beta }$ is the component of the metabolic exponent that evolves from the primary (pre‐mass‐rescaling) selection of metabolism, and ${\hat{\beta }}_{w}$ is the component that evolves from mass‐rescaling selection (Witting, 2017). Then, as ${\hat{\beta }}_{w}=-1/2d$ (Witting, 2017), we may define the dependence of mass‐specific metabolism on the β‐dependent minimum mass from the primary relation

(13) $$\partial \ln\beta_{\beta }/\partial \ln{\underline{w}}_{\beta }^{*}={\hat{\beta }}_{\beta },$$

where ${\hat{\beta }}_{\beta }=\hat{\beta }+1/2d$, with the primary exponent (${\hat{\beta }}_{\beta }$) declining monotonically toward zero with an increase in ${\underline{w}}_{\beta }^{*}$, from an initial value (${\hat{\beta }}_{\beta ,0}$) at the lower mass limit (${\underline{w}}_{\beta }^{*}=\underline{w}$) that defines the transition between the replicator with no metabolism and the smallest self‐replicator with an intrinsic metabolism.

To transform Equations (11) and (13) into an initial selection gradient for the joint evolution of metabolism and mass, we note from Witting (2017) that some of the allometric correlations among the different traits cancel each other. This allows for a simpler expression of the self‐replication ($\lambda =pt_{r}\epsilon /\overset{´}{\beta }w$, Equation (1)) that generates the frequency‐independent selection on metabolism and mass. With the metabolic‐rescaling component of survival being proportional to the primary selection of mass‐specific metabolism for all allometric solutions ($p_{\beta }\propto \beta_{\beta }\propto 1/t_{\beta }$, Witting, 2017), it follows that $pt_{r}$ scales as $p_{w}t_{w,r}$. Then, given mass invariance for $p_{w}$ and $\overset{´}{\beta }$ (Witting, 2017), we have $\lambda \propto t_{w,r}\epsilon /w$, that reduces to $\lambda \propto \beta_{\beta }/w$ given $\epsilon \propto \beta \propto \beta_{\beta }\beta_{w}$, $t_{w,r}\propto 1/\beta_{w}$, and $\hat{\alpha }=0$ initially. And with r=lnλ, we obtain the following fitness profile

(14) $$r^{*}\propto \left({\hat{\beta }}_{\beta }-1\right)\ln{\underline{w}}_{\beta }^{*},$$

and selection gradient

(15) $$\partial r/\partial \ln{\underline{w}}_{\beta }^{*}={\hat{\beta }}_{\beta }-1$$

on the β‐dependent minimum mass.

### 3.3 The evolution of virus

Before we use Equation (15) to predict the evolution of self‐replicating cells with internal metabolism, let us consider the other side of the coin that is an evolutionary dead end that maintains molecular replicators with no metabolism as the selection attractor at the cost of the evolution of self‐replication, metabolism and mass.

For a virus‐like replicator with no internal metabolism to persist as a selection attractor, it is required that the self‐replication selection at the potential transition between the molecular replicator with no metabolism and the smallest self‐replicator is selecting for the molecular replicator at the cost of a self‐replicator with an internal metabolism. This selection occurs whenever the maximum exponent ${\hat{\beta }}_{\beta }={\hat{\beta }}_{\beta ,0}$ of Equation (15) is smaller than one. The selection gradient is then negative with constant selection for a joint decline in mass and mass‐specific metabolism.

Unlike selected self‐replicators, these replicators cannot increase replication by an increase in mass‐specific metabolism. This is because the energetic demands for the mass of the offspring increases linearly with mass, while the metabolism that generates the required energy can only increase sublinearly with mass. The replicators may instead increase their replication rate by a mass that evolves toward zero, with the side effect that they are shutting down their metabolic processes. The final attractor

(16) $$\beta^{**}=0\wedge {\underline{w}}^{\underline{\underline{*}}\underline{*}}$$

is the molecular replicator with no internal metabolism, no cell, practically no mass, no self‐replication, and a replication that is driven by an extrinsic source of metabolism (for asterisk notation, see Table 3).

**Table 3:** Selection attractors. The asterisk (**) superscripts and main parameters of the selection attractors that evolve from a gradual unfolding of population‐dynamic feed‐back selection from density‐dependent interactive competition. The bar notation on the left ∗ describes the selection status of ϵ, β and α and that of the right ∗ the selection status of w. Underlined asterisks denote a downward minimum or selection constraint, overlined an upward constraint, and unlined no constraint

∗∗	ϵ	β	α	w	ιψ	${\hat{\beta }}_{\beta }$	Description	Cells	Reproductive unit
$\underline{\underline{*}}\underline{*}$	0	0	$\underline{\alpha }$	$\underline{w}$	0	–	Replicator; no cell; minimum mass	0	Asexual
$\underline{**}$	ϵ	β	$\underline{\alpha }$	${\underline{w}}_{\beta }$	0	1	Self‐replicating cell; β‐dep. minimum mass	1	Asexual
$*\underline{*}$	ϵ	β	α	${\underline{w}}_{\beta }$	0–1	$1-\bar{\iota }\psi$	as $\underline{**}$, but $\alpha >\underline{\alpha }$ and interactive selection	1	Asexual
$\bar{*}*$	$\bar{\epsilon }$	$\bar{\beta }$	$\bar{\alpha }$	w	1	0–1	Multicellular animal; upward constrained ϵ	≫1	Sexual; male/female pair
∗∗	ϵ	β	α	w	$\frac{4d-1}{2d-1}$	0–1	as $\bar{*}*$, but unconstrained ϵ	≫1	Sexual; cooperative
$\bar{**}$	$\bar{\epsilon }$	$\bar{\beta }$	$\bar{\alpha }$	$\bar{w}$	≫1	–	as $\bar{*}*$, but upward constrained mass	≫1	Eusocial; stable colony
$*\bar{*}$	ϵ	β	α	$\bar{w}$	→∞	–	as $\bar{**}$, but unconstrained ϵ	≫1	Eusocial; increasing colony

Molecular replicators with no metabolism and practically no mass may thus not only be the potential ancestor for more complex self‐replicators with an internal metabolism, but they may also be selection attractors of self‐replication selection itself.

Although extremely small, viruses are likely significantly larger than the replicating molecules that were formed prior to the evolution of self‐replicating cells with an internal metabolism. These relatively large masses of viruses are expected to be selected by an energy‐assisted replication that is fueled by the metabolism of their host. Because of this increased mass, the maximum metabolic exponent (${\hat{\beta }}_{\beta ,0}$) may be smaller in viruses than in an initial molecular replicator that was formed by other processes than replication. It is thus quite likely that viruses have masses that are situated at a joint attractor, where they are selected not only by energy‐assisted‐replication, but also by the self‐replication selection that occurs in viruses that mutate to some initial forms of intrinsic metabolism. Having no metabolism and a time‐scale that depends on the metabolism of their host, viruses are selected beyond the allometric model in Witting (2017).

## 4 MINIMUM SELF‐REPLICATING CELLS

Let us now consider what happens when the maximum exponent for the primary selection of mass‐specific metabolism (${\hat{\beta }}_{\beta ,0}$) is larger than one.

### 4.1 The initial metabolism and mass

Given replicating molecules that are smaller than viruses, it is likely that at least some of these will have a maximum primary exponent that is larger than one (${\hat{\beta }}_{\beta ,0}>1$). The only thing that is required is the potential for heritable mutations toward metabolic pathways of self‐replication, where the attachment of catalysts to an initial self‐replicator is causing an increase in mass‐specific metabolism that is about proportional to the increase in mass [Recall that the $\hat{\beta }={\hat{\beta }}_{\beta }-1/2d$ exponent that defines the final relationship between β and *w* is smaller than the ${\hat{\beta }}_{\beta }$ exponent in Equation (15)]. When this is the case, we find from Equations (13) and (15) that the selection attractor is an equilibrium β‐dependent minimum mass (${\underline{w}}_{\beta }^{\;\underline{**}}$), that is defined by a primary exponent of unity

(17) $${\hat{\beta }}_{\beta }\left({\underline{w}}_{\beta }^{\;\underline{**}}\right)=1.$$

**Figure 1:**
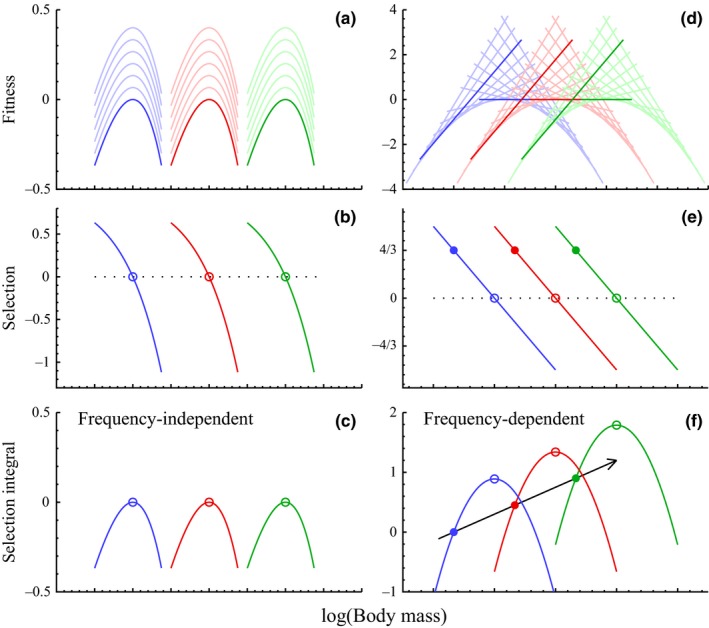
Body mass selection. Fitness profiles/landscapes (a and d; $r_{i}^{*}=f\left(w_{i}\right)$, intrapopulation variation [subscript i] in populations that are in dynamic equilibrium), selection gradients (b and e; $\partial r_{i}^{*}/\partial \ln w_{i}{|}_{w_{i}=w}$; across population variation [no subscript]), and selection integrals (c and f; $\int (\partial r_{i}^{*}/\partial \ln w_{i}{|}_{w_{i}=w})\;d\ln w$; across population variation) for the frequency‐independent selection of the physiology (a–c; calculated from model in Section A1) and the density–frequency‐dependent selection of interactive competition (d–f; from equations (27) to (29), given 2D interactions). Selection integrals look like fitness landscapes for physiological selection, but they cannot be visualized from the fitness landscape of density‐dependent interactive competition. Equilibrium attractors are shown by open circles (different colors different attractors), and unconstrained selection by interactive competition has steady state attractors (solid circles) with a selection integral evolution (black arrow) that is driven by a selected exponential increase in net energy and mass (Witting, 1997). The multiple fitness profiles per color represent populations with different average variants, with clear colored curves having average variants at evolutionary equilibrium or steady state

As this is an equilibrium of frequency‐independent selection, we expect a straightforward relationship between the fitness profile and the selection integral, as illustrated in Figures 1a–c.

We may thus conclude that when the maximum exponent for the primary selection of mass‐specific metabolism is larger than one (${\hat{\beta }}_{\beta ,0}>1$), it follows that the net energy that is obtained by the increase in mass‐specific metabolism increases stronger than linearly with mass, and this will generate an increase in the self‐replication rate with mass. This selection for an increase in metabolism and mass will continue until the exponent (${\hat{\beta }}_{\beta }$) for primary selection has declined to unity, and the net energy that is generated from extra metabolism is exactly balancing the selection of the quality–quantity trade‐off (Equation (11)).

### 4.2 Life‐history

In relation to the life‐history of these minimum‐sized self‐replicators, we note that for cases where the maximum ${\hat{\beta }}_{\beta ,0}$ exponent is only slightly larger than one, we might expect the evolution of self‐replicators with simple metabolic pathways and no cell. But for larger ${\hat{\beta }}_{\beta ,0}$ exponents, we can expect selection for the formation of a cell‐like structure with an increased mass and an internal metabolism.

As it is the formation of the cell that is structuring the metabolic pathways, and as these self‐replicators are selected to have the minimum possible mass that is required for their metabolism, they are selected to have a single cell only. And with a primary selection exponent (${\hat{\beta }}_{\beta }$) of unity for mass‐specific metabolism, the selection attractors are so small that their metabolic pathways are incomplete in the sense that it is biochemically possible to increase the metabolic efficiency per unit mass.

A ${\hat{\beta }}_{\beta }$ exponent of unity implies also that the body mass exponent for resource handling ($\hat{\alpha }$) is predicted to be zero (Witting, 2017). This indicates that these self‐replicating cells have a passive handling of their resources. And as the selection attractors will have no significant resource bias from interactive competition, they have frequency‐independent selection for asexual reproduction (Witting, 1997, 2002a).

### 4.3 Allometries

With the dependence of mass‐specific metabolism on mass being different for different metabolic pathways, and the dependence evolving with the evolving self‐replicator, we can expect low‐energy self‐replicators with different minimum masses because of adaptations to a variety of niches and metabolic pathways. And with these attractors being predicted as the smallest group of self‐replicating cells, they would resemble prokaryotes. The predicted allometric exponents are given by the ${\hat{\beta }}_{\beta }=1$ rows in Table 2. For behavior in three spatial dimensions, this implies a mass‐specific metabolism and a population‐dynamic growth rate that increase to the 5/6 power of mass.

Metabolic exponents for prokaryotes are inherently difficult to define and estimate, not only because metabolism and mass are strongly dependent on an active or inactive physiological state, but also because of non‐negligible errors associated with most estimates of mass. By correcting estimated exponents to their reduced major axis values to account for uncertain estimates of mass, DeLong et al. (2010) estimated empirical exponents in heterotrophic prokaryotes around 0.84 (0.96 ± 0.18 for active; 0.72 ± 0.07 for inactive) for mass‐specific metabolism and 0.73 for the rate of population growth. While these estimates may be preliminary, they agree with the predicted value of 0.83. This indicates that the body masses of prokaryotes may indeed evolve from a positive dependence of mass‐specific metabolism on the mass of the self‐replicating cell.

## 5 INTERACTING SELF‐REPLICATING CELLS

Minimum‐sized self‐replicators that are larger than the attractor of Equation (17) will have an increased mass‐specific metabolism and more net energy available. But they cannot be selected exclusively by the dependence of mass‐specific metabolism on mass.

### 5.1 Evolution of interactive competition

As there seems to be no physiological constraint that will generate frequency‐independent selection for a general increase in mass (see Appendix 1), let us examine how the emergence of a resource bias from interactive competition is affecting the selection of metabolism and mass.

This frequency‐dependent selection reflects that the larger individuals in a population may dominate the smaller individuals during competitive encounters, at least when other things are equal. This is expected even for the passive type of behavior that we may expect in simple self‐replicators, where larger individuals will have more kinetic energy than smaller individuals and thus also a higher probability of winning a competitive encounter. This will create a bias ($\Delta \mu_{i}=\mu_{i}-\mu$) in the fitness cost (μ) of a competitive encounter, where $\Delta \mu_{i}$ is the cost of the *i*th variant relative to the average cost (μ) in the population. With a symmetrical cost bias on logarithmic scale, and ψ being the gradient that measures the fitness cost per unit interference on log scale across the body mass variation in the population, we find that the differentiation in the cost of interference per unit interference is

(18) $$\Delta \mu_{i}=\psi \ln\left(w/w_{i}\right).$$

Here, it is the mass ($w_{i}$) of an individual (*i*) relative ($w_{i}/w$) to the average mass (*w*) that defines the ecological dominance of the individual in the population. This translates into the fitness profile

(19) $$r_{i}^{*}\propto \left({\hat{\beta }}_{\beta }-1\right)\ln{\underline{w}}_{\beta ,i}^{*}+\psi {\bar{\iota }}^{*}\ln\left({\underline{w}}_{\beta ,i}^{*}/{\underline{w}}_{\beta }^{*}\right)$$

for the potential evolution of minimum masses ${\underline{w}}_{\beta }^{*}$, with the cost of interference being positively dependent on the level of intraspecific interference (ι), which is maintained at its maximum by the selected β‐dependent minimum mass, where ${\bar{\iota }}^{*}=\left(\gamma_{\iota }/\gamma \right)\ln\left({\overset{´}{\epsilon }}_{0}/{\underline{w}}_{\beta }^{*}\right)$ from equation 83 in Witting (2017) ($\gamma_{\iota }$: density dependence of interference; γ: overall density dependence; ${\overset{´}{\epsilon }}_{0}$: a measure of net energy on the per‐generation time‐scale; bar on ${\bar{\iota }}^{*}$ denoting maximum interference as defined by the β‐dependent minimum mass, ${\underline{w}}_{\beta }^{*}$).

The selection gradient

(20) $$\partial r^{*}/\partial \psi ={\bar{\iota }}^{*}\left(\ln{\underline{w}}_{\beta ,i}^{*}/{\underline{w}}_{\beta }^{*}\right)$$

on the cost gradient of Equation (19) is positively related to the level of interference, with disruptive selection for a larger ψ in the individuals that are larger than the average, and a smaller ψ in the individuals that are smaller than the average. This reflects a selection battle, where the larger than average individuals are selected to monopolize the resource during encounters, and the smaller than average individuals are selected to avoid the monopolization of the larger individuals. While the avoidance of smaller individuals may limit the average gradient to some degree, the energetic dominance of the larger individuals sets a natural upper limit to this avoidance. It is thus the monopolization component that will dominate the evolution of the cost gradient, with mass being selected as a trait that is used to dominate other individuals during interactive encounters.

### 5.2 Initial interactive selection of mass

To describe this interactive selection of mass, from the fitness profile of Equation (19), we obtain the selection gradient across the intraspecific variation in the β‐dependent minimum mass

(21) $${\left.\frac{\partial r_{i}^{*}}{\partial \ln{\underline{w}}_{\beta_{i}}^{*}}\right|}_{{\underline{w}}_{\beta_{i}}^{*}={\underline{w}}_{\beta }^{*}}=\psi {\bar{\iota }}^{*}+{\hat{\beta }}_{\beta }-1.$$

The evolution of an interacting self‐replicator, and a ${\hat{\beta }}_{\beta }$‐exponent that is smaller than one, is thus dependent upon the evolution of a maximum resource bias that is larger than zero ($\psi {\bar{\iota }}^{*}>0$). The evolution of such a gradient will generate a selection attractor

(22) $${\hat{\beta }}_{\beta }\left({\underline{w}}_{\beta }^{*\underline{*}}\right)=1-\psi {\bar{\iota }}^{*},$$

where a β‐dependent minimum mass is defined by an exponent for the primary selection of mass‐specific metabolism that is smaller than unity.

The evolution of this minimum mass can continue along a gradient where the maximum cost bias is increasing toward unity. This reflects a population‐dynamic feed‐back selection that is unfolding as the selected increase in the average net energy (Equation (2)) is generating extra population growth, with a higher population density with increased interference among the individuals in the population. The feed‐back selection, however, is not yet fully developed, and body mass selection is still dependent upon the frequency‐independent dependence of mass‐specific metabolism on minimum mass.

### 5.3 Life‐history

From Equations (13), (17) and (22), we find that the interacting self‐replicators are larger than self‐replicators with no interactions and that they increase in size with an evolutionary increase in the maximum resource bias ($\psi {\bar{\iota }}^{*}$) of interactive competition. As prokaryotes, they are selected to have the minimum possible mass that is required for their metabolic functioning, and they are thus selected to be unicellular self‐replicators. And being predicted as a second size class of the unicellular attractor beyond replicating molecules, we expect the interacting self‐replicating cells to resemble larger unicells like protists and protozoa.

We note also that the frequency‐dependent selection from the unfolding feed‐back is dependent on net energy that is generated from an independent selection on resource handling. This is because of the $\hat{\epsilon }=\hat{\alpha }+{\hat{\beta }}_{\beta }=1$ relationship from Equation (25) in Witting (2017). This implies that the evolution is dependent upon the evolution of a proportional relationship between mass and the pre‐mass‐rescaling component of net energy. And with the ${\hat{\beta }}_{\beta }$ exponent being smaller than one, it follows that the exponent for resource handling ($\hat{\alpha }$) is selected to be larger than zero.

It thus seems that an evolutionary increase in resource handling is needed to maintain the frequency‐dependent selection of interactive competition that is required for an evolutionary increase in the mass of the interacting self‐replicating cell. Hence, the evolution of protozoa‐like cells seems to require both increased resource handling and an interactive competition that generates some bias in the resource distribution over mass. Protozoa and other larger unicells are thus predicted to have a more organized and developed resource handling and interactive behavior than the individuals of prokaryotes.

As these larger unicellular self‐replicators are predicted to have a maximum resource bias that is smaller than unity ($\psi {\bar{\iota }}^{*}<1$), it follows that their selection for major life‐history transitions is dominated by the frequency‐independent selection of their physiology (Witting, 1997, 2002a, 2008). This implies that they are selected to have asexual reproduction. But as the transition boundary for the frequency‐dependent selection of multicellular organisms with sexual reproduction lies at a maximum resource bias of unity (Witting, 1997, 2002a, 2008 and next section), we may also expect that some of the larger interacting self‐replicators with the strongest resource bias can be selected to show some initial traces of both multicellularity and sexual reproduction.

### 5.4 Allometries

To examine the selection of allometries, recall that the variation in body mass across populations of interacting self‐replicating cells are predicted to evolve from the co‐evolutionary continuum in net energy and behavioral interactions that will make the maximum cost bias ($\psi {\bar{\iota }}^{*}$) increase from zero to one. Hence, from Equation (22), we predict a joint evolutionary increase in mass and the primary selected mass‐specific metabolism, with the exponent for the primary selection of mass‐specific metabolism (${\hat{\beta }}_{\beta }$) declining from one to zero. So instead of having a continued increase in mass‐specific metabolism with mass, as predicted for the minimum self‐replicator, we find from Table 2 that interacting self‐replicators are predicted to have an initial increase in mass‐specific metabolism with mass (5/6 exponent in 3D and 3/4 exponent in 2D), that will decline with mass first to a mass invariance, and then to a negative correlation with an exponent that approaches −1/6 in 3D, and −1/4 in 2D, as the dependence of mass‐specific metabolism on mass is vanishing with the evolutionary increase in mass.

By excluding the four smallest protozoa with exceptionally high metabolic rates from the data of Makarieva et al. (2008), and by least‐squares fitting a third‐degree polynomial to the remaining data for inactive protozoa (*n* = 48), Witting (2017) obtained point estimates of the body mass exponent for mass‐specific metabolism that declined from 0.61 across the smallest [logw(kg)=−13.5], over zero across intermediate [logw(kg)=−11], to a minimum of −0.20 among the largest protozoa [logw(kg)=−8.0, see Figure 4]. This change indicates that protozoa may be selected by an interactive feed‐back selection that is unfolding with an increase in the maximum cost bias.

## 6 MULTICELLULAR ANIMALS

**Figure 2:**
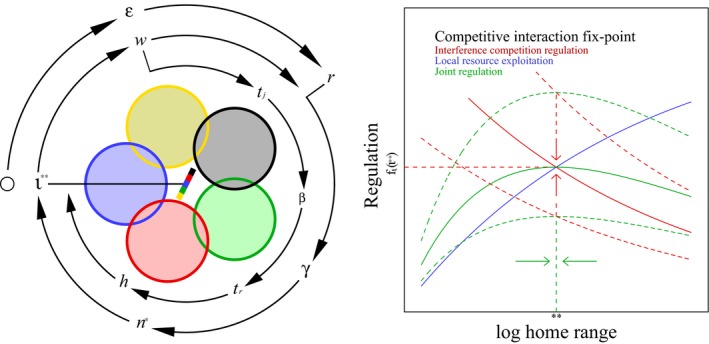
Population‐dynamic feed‐back selection. Left: Feed‐back diagram, with symbols that relate to the population average, and colored circles that symbolize individual home ranges in two‐dimensional space with interactive competition in zones of overlap. Winners (dominating color) monopolize resources, and this generates a body mass biased resource access that is proportional to the slope of the multicolored bar in centrum, with the invariant interference (ι**) of the selection attractor determining the evolution of this bias. The black o to the left represents the origin of self‐replication, and selection for an exponential increase in net energy (ϵ) maintains a relatively high r and continued feed‐back selection for an exponential increase in mass. The attractor of the feed‐back is given by the outer ring of symbols (r: population growth →γ: density regulation $\to n^{*}$: population abundance →ι: interference level →w: selection on body mass →r: population growth). Selection for a change in mass initiates the inner loop of mass‐rescaling selection (w: mass change → $t_{j}$: juvenile period →β: metabolic rate $\to t_{r}$: reproductive period →h: home range →ι: interference; see Witting (2017) for details). Both loops adjust to the invariant interference of the competitive interaction fix‐point, which evolves by the selection attractor of density regulation in the right plot: The home range of optimal density regulation (**) is defined by the joint regulation ($f_{\iota }f_{s}$; green curves) of interactive competition ($f_{\iota }$; red curves) and local resource exploitation ($f_{s}$; blue curve). As the optimal home range (equation 14 in Witting, 2017) is independent of the feed‐back between the population abundance and the interactive selection on mass, and as the level of interference is dependent on abundance, it follows that the density regulation of interference competition is adjusted by body mass selection to a joint selection attractor (solid red and green curves), where regulation at the home range optimum coincides with the regulation [$f_{\iota }\left(\iota^{**}\right)$] of the competitive interaction fix‐point ($\iota^{**}$) for the selection attractor on mass

A next transition in the evolution of metabolism and mass occurs when the maximum cost bias of interference competition evolves beyond unity ($\psi {\bar{\iota }}^{*}>1$). This will cause the unfolding of a fully developed population‐dynamic feed‐back selection (Figure 2, left) that selects for organisms that are larger than the minimum that is required for their metabolic functioning. This makes the ${\hat{\beta }}_{\beta }$ exponent functionally independent of mass, and it allows for an in principle independence between the evolution of mass, resource handling, and mass‐specific metabolism. The three traits, however, will be interlinked by mass‐rescaling and population‐dynamic feed‐back selection, where the selection of mass is dependent on the net energy that is generated by resource handling and metabolic pace.

### 6.1 Competitive interaction fix‐points

With a body mass that is selected beyond the minimum that is required for a given mass‐specific metabolism, the intrinsic dependence of mass‐specific metabolism on mass is vanishing. The fitness profile on body mass is thus reducing to

(23) $$r_{i}^{*}\propto \psi \iota^{*}\ln\left(w_{i}/w\right)-\ln w_{i},$$

with the following selection gradient

(24) $${\left.\frac{\partial r_{i}^{*}}{\partial \ln w_{i}}\right|}_{w_{i}=w}=\psi \iota^{*}-1.$$

This implies that the equilibrium attractor of population‐dynamic feed‐back selection is a competitive interaction fix‐point

(25) $$\iota^{\bar{*}*}=1/\psi ,$$

with an invariant level of interference competition as the population condition for a neutrally stable selection attractor on mass (Witting, 1997, 2003). This interference is exactly so high that the increased reproductive potentials of the smaller individuals are balanced against the resource monopolization of the larger individuals so that fitness is similar across variation in mass.

The $\bar{*}*$‐attractor of Equation (25) is the result of a stable net energy $\bar{\epsilon }$ that is upward constrained for some reason, with overline bar denoting the upper limit. The ∗∗‐attractor for unconstrained selection with an exponential increase in ϵ (Equation (2)) is characterized by a somewhat higher level of invariant interference

(26) $$\iota^{**}=\left(4d-1\right)/\left(2d-1\right)\psi ,$$

and an exponentially increasing mass (Witting, 1997, 2003).

The competitive interactions fix‐points of Equations (25) and (26) are based on an unconstrained body mass, where the attracting mass ($w^{\bar{*}*}$ & $w^{**}$) is an energetic buffer that is selected to absorb the energy that is initially selected into enhanced reproduction and population growth. If the mass evolves to an upper bound ($\bar{w}$), it follows that this energy can no longer be absorbed by the selection of mass, with the result that the population density and the level of interference competition evolves to a much higher stable ($\iota^{\bar{**}}\gg 1/\psi$) or increasing ($\iota^{*\bar{*}}\to \infty$) level, dependent upon a stable or increasing net energy (Witting, 1997, 2003).

The right plot in Figure 2 shows how the competitive interaction fix‐point is co‐evolving with the selection of the optimal density regulation. The latter is adjusting the home range to optimize the foraging behavior as a balance between the cost of interference competition and the cost of the local resource exploitation of the individual (Witting, 2017). This shows that the density regulation of interference [$f_{\iota }\left(\iota \right)$] is adjusted by the population‐dynamic feed‐back selection to a joint selection optimum, where the interference regulation of the home range optimum is coinciding with the regulation [$f_{\iota }\left(\iota^{**}\right)$] of the competitive interaction fix‐point (e.g., $\iota^{**}$).

### 6.2 Interactive selection of mass

It is possible to visualize the mass attractor of population‐dynamic feed‐back selection by including the population‐dynamic feed‐back of density‐dependent interference competition (equation 83 in Witting, 2017) into the equations. The fitness profile (Figure 1d) on body mass is then

(27) $$r_{i}^{*}=\ln\left[w/w_{i}\right]\left[\psi \gamma_{\iota }/\gamma \ln\left({\overset{´}{\epsilon }}_{0}/w\right)-1\right],$$

and the selection gradient (Figure 1e)

(28) $${\left.\frac{\partial r_{i}^{*}}{\partial \ln w_{i}}\right|}_{w_{i}=w}=\left(\psi \gamma_{\iota }/\gamma \right)\ln\left({\overset{´}{\epsilon }}_{0}/w\right)-1.$$

The latter equation is then integrated across the potential evolution of the average variant in the population to obtain the selection integral

(29) $$\int \left({\left.\frac{\partial r_{i}^{*}}{\partial \ln w_{i}}\right|}_{w_{i}=w}\right)d\ln w\propto \ln w\left[\left(\psi \gamma_{\iota }/\gamma \right)\ln\left({\overset{´}{\epsilon }}_{0}/\sqrt{w}\right)-1\right],$$

which illustrates the evolutionary attractor in Figure 1f. This selection has no absolute measure of relative fitness and no concept of an increase in average fitness. The result is a fitness landscape (profile) that has no direct visualization with the attractor of the selection integral (compare Figure 1d,f).

### 6.3 Life‐history

The transition in the interactive competition to a maximum cost gradient above unit is not only selecting for a transition from self‐replicators with the minimum masses that are required for their metabolic functioning, to larger organisms where energy is selected into mass to enhance the competitive quality of the individual. With a mass that is no longer selected to the minimum that is required to sustain metabolism, there is no longer direct selection for the existence of a single metabolic compartment only (one cell).

In the absence of direct selection for single‐celled individuals, co‐operation at a higher multicellular level may trade‐off fitness at the cellular level to enhance the overall fitness of the organism (Buss, 1987; Michod, 1996, 1997, 1999; Michod & Roze, 2001). The increased functionality of resource handling, interactive behavior and resource transportation that can be obtained from a division of a single large cell into a multitude of co‐operating cells with smaller masses, is then expected to select for the emergence of multicellular organisms.

The selection of other life‐history traits is also dependent on the level of interference in the population, with the transitions between the different competitive interaction fix‐points selecting for major life‐history transitions (Witting, 1997, 2002a, 2008). The increase in the maximum cost gradient above unity is likely to induce selection for a soma with senescence, where it is beneficial to take energy from self‐repair and use it in interactive competition (Witting, 1997, 2008). And it is also selecting for the evolution of sexual reproduction between a male and female individual, where the male is specializing in interactive competition at the cost of self‐replication, and the female is sharing her genome in the offspring with the male in order to attract the competitively superior males (Witting, 1997, 2002a, 2008). The result is interactive competition that is generating frequency‐dependent selection for the evolution of sexual reproduction with a diploid genome.

This selection for a reproducing unit with specialized multicellular individuals and sexual reproduction is unfolding to higher levels of organization with an increase in the interactive competition in the population (Witting, 2002a). Where the interactive competition of the energetically constrained mass attractor ($w^{\bar{*}*}$) is selecting for pairwise sexual reproduction, the increased interference at the evolutionary steady state ($w^{**}$) is selecting for cooperative reproduction where a single or a few offspring workers are enhancing the interactive quality of the sexually reproducing pair. And the potentially much higher interference at the upward constrained mass attractors (${\bar{w}}^{*\bar{*}}$ or ${\bar{w}}^{\bar{**}}$) selects for an eusocial colony with thousands of interacting offspring workers; with the energetically constrained attractor ($\bar{**}$) having a stable colony size, while the increasing energy of the energetically unconstrained attractor ($*\bar{*}$) is selected into a continued increase in colony size (Witting, 2002a).

Owing to a diminishing return in the interactive quality that the reproducing unit can gain from sexual reproduction between a female and an increasing number of males, these co‐operatively and eusocially reproducing units have active selection for pairwise sexual reproduction and offspring workers at the cost of higher levels of sexual reproduction with more than two individuals involved (Witting, 2002a). And where the pair‐bond in eusocial termite‐like species selects for a diploid genome, the absence of a pair‐bond in eusocial hymenoptera‐like species is selecting for a male‐haploid‐female‐diploid genome (Witting, 1997, 2007).

### 6.4 Allometries

With primary selection on metabolism and resource handling being independent of the selection of mass, the allometric exponents for multicellular animals may in principle take any of the values in Table 2. What is essential is the underlying cause for the variation in the mass of the species that are being compared in the allometric correlation.

If the primary cause is selection on metabolic pace, we expect an exponent for the primary selection of mass‐specific metabolism around one, and a mass‐specific metabolism with a 5/6 exponent in 3D, and a 3/4 2D exponent. If instead, we are examining species that have diversified and adapted to a broad spectrum of niches, the primary cause is variation in the handling and/or densities of the underlying resources. The exponent for the primary selection of mass‐specific metabolism is then around zero, and we have Kleiber scaling with −1/4 exponents in 2D, and −1/6 exponents in 3D.

Table 3 lists all the selection attractors on mass together with a brief description of the predicted life histories, ranging from replicating molecules to the eusocial colony of the multicellular animal. The conditions that determine the attractor of an evolutionary lineage at a given point in time are the selection and constraint status of mass‐specific metabolism (β), resource handling (α) and mass (*w*), and the associated resource bias in the population (ιψ).

## 7 SELECTION OF MAJOR TAXA

**Figure 3:**
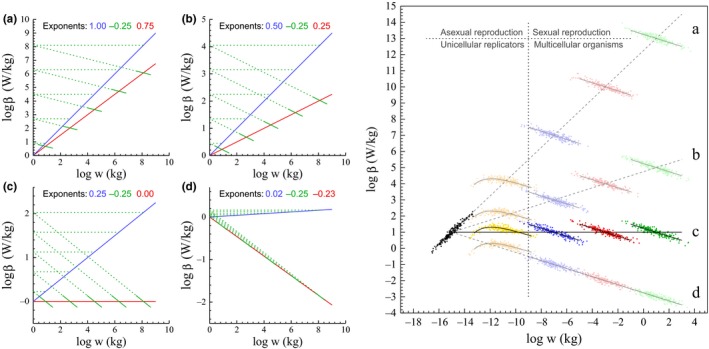
Selection of major taxonomic groups. Given 2D interactions, the four plots to the left illustrate the mechanistic relationship between the metabolic‐rescaling (blue), mass‐rescaling (green), and final (red) allometries (exponents given by colored numbers), with evolving taxa illustrated by the different colored species distributions in the right‐hand plot (each dot is a species). Left plots: The primary selection of metabolism generates the metabolic span of the blue lines based on the different metabolic‐rescaling exponents (${\hat{\beta }}_{\beta }$, blue numbers) in plots (a–d). The associated selection of mass generates the span in mass, with the solid green lines showing the local mass‐rescaling (within an evolutionary lineage or across species in taxonomic groups) that makes the final allometric relationship evolve along the red lines. The dotted green lines show that the scaling from the primary selection of metabolism (blue) is the scaling of the intercepts of local mass‐rescaling (solid green) on the final allometry (red). Prokaryotes (black dots right‐hand plot) are proposed to evolve body mass variation from the primary selection of metabolism (mechanism (a), ${\hat{\beta }}_{\beta }=1$). Protozoa (yellow dots) to evolve by a gradual shift from (a) to (d), with the selection of net energy changing from selection on metabolism to selection on resource handling/resource availability (${\hat{\beta }}_{\beta }=1\to 0$). And taxa of multicellular animals to evolve mass variation primarily from speciation across ecological niches (mechanism (d), ${\hat{\beta }}_{\beta }\approx 0$). The latter taxa (blue, red, and green dots in right plot) will differentiate along lines (a–d) dependent upon the underlying mechanism (left plots a–d) that selects the mass variation across major taxa. The most likely mechanism is (c), where ${\hat{\beta }}_{\beta }=1/2d$ and mass‐specific metabolism evolves along an upper bound

The relationships between metabolic‐rescaling, mass‐rescaling, and the final allometry are shown in the left plots in Figure 3, for four selections where all (${\hat{\beta }}_{\beta }=1$) to none (${\hat{\beta }}_{\beta }=0$) of the variation in net energy is generated from primary selection on mass‐specific metabolism. This illustrates (1) how a positive scaling between mass‐specific metabolism and mass can be selected in prokaryote‐like self‐replicators from primary selection of metabolism (${\hat{\beta }}_{\beta }=1$), (2) how a decline in the exponent may evolve in protozoa and protists from a change toward primary selection on resource handling (${\hat{\beta }}_{\beta }=1\to 0$), and (3) how the body mass variation in taxa of multicellular animals can evolve from diversification across ecological niches (${\hat{\beta }}_{\beta }\approx 0$). Yet, selected variation in mass‐specific metabolism will also generate body mass variation in multicellular animals, and we may therefore ask whether the evolution of metabolism and mass is interlinked in a macroevolutionary pattern with different allometric exponents within and across the major taxa of multicellular animals?

The local mass‐rescaling of the solid green lines in the left plots in Figure 3 can be seen as species distributions of different taxa. The red lines of the different plots will then represent different routes for an evolutionary divergence between the major taxa, with the possible macroevolutionary routes being dependent on the natural selection of metabolic pace.

This interpretation is illustrated in the right‐hand plot, together with the transition to the sexually reproducing multicellular animal, which is predicted to lie at the lower boundary where interactive competition can select for a larger than minimum‐sized organism, which is also the boundary of the within taxa −1/2*d*‐power scaling of mass‐specific metabolism with mass.

If the variation across major taxa evolves with a ${\hat{\beta }}_{\beta }$ exponent around zero, we should see a single 1/2*d*‐like scaling across all larger species (case d). But this is not our first expectation as it would imply a general absence of natural selection on metabolic pace. A ${\hat{\beta }}_{\beta }$ exponent around unity (case a) is also unlikely, as it would imply that all the mass variation between major taxa would be induced by selection differences in metabolism, while the within taxa variation would evolve by differences in resource handling and availability. A more likely base‐case could be ${\hat{\beta }}_{\beta }=1/2$ (case b), where natural selection on pace and handling is almost equally important at the scale of the major taxa.

**Figure 4:**
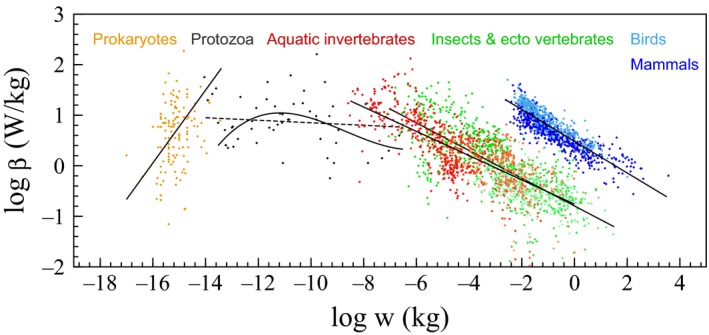
Heterotroph organisms. Macroevolutionary relationship between mass and minimum mass‐specific metabolism among heterotroph organisms. Data from Makarieva et al. (2008), with RMA lines from DeLong et al. (2010) for prokaryotes and protozoa, and least‐squares lines for other taxa. Prokaryotes: $\hat{\beta }=0.72$, *n* = 123, for passive ($\hat{\beta }=0.96$ for active); protozoa: $\hat{\beta }=-0.03$, *n* = 52; aquatic invertebrates: $\hat{\beta }=-0.24$, *n* = 808; insects & ectotherm vertebrates: $\hat{\beta }=-0.27$, *n* = 982; birds & mammals $\hat{\beta }=-0.31$, *n* = 948. A least‐squares fitted third‐order polynomial is also shown for protozoa, excluding the left‐top four species

But we have already predicted that the metabolic pathways should be fully developed at the evolutionary transition to the multicellular animal, and this implies a ${\hat{\beta }}_{\beta }$ exponent around 1/2*d* (case c). Macroevolution would then proceed along an upper bound on mass‐specific metabolism, where an evolutionary increase in mass would require a primary increase in resource handling and/or resource availability. This would generate a mass‐rescaling with an allometric downscaling of mass‐specific metabolism and, if handling increases sufficiently slowly, we should expect a subsequent increase in mass‐specific metabolism due to primary selection on biotic pace. The overall macroevolution of mass‐specific metabolism would then proceed along a metabolic bound, while evolutionary diversification across niches would generate −1/2*d*‐power scaling within large bodied taxa that expand in species numbers across ecological niches. Patterns of mass‐specific metabolism within and across major taxa from prokaryotes to mammals have been studied by DeLong et al. (2010), Kiørboe and Hirst (2014), Makarieva, Gorshkov, and Bai‐Lian (2005), and Makarieva et al. (2008), with the overall pattern (Figure 4) resembling the theoretical expectation of ${\hat{\beta }}_{\beta }=1/2d$ (Figure 3, right, c).

## 8 DISCUSSION

As the proposed selection explains the evolution of complete life histories from a few basic principles, it deals with the simultaneous selection of a diverse set of life‐history traits that include metabolism, mass, allometric exponents, reproduction, survival, life periods, abundance, home range, cellularity, senescence, and sexual reproduction. These rather complex predictions emerge from the interrelatedness of a multitude of equations in several publications on MR (Witting, 1997, 2002a, 2008, 2017), and the resulting theory may therefore appear unnecessary complex at first. But it is in fact extremely parsimonious because all of the major transitions in the life‐history and allometric exponents are selected by the primary selection that is necessary for the evolution of metabolism and mass.

The complete evolutionary unfolding from replicating molecules to multicellular animals with sexual reproduction is, in essence, explained by four simple principles. These are (1) natural selection by differential self‐replication in populations, (2) conservation of energy, (3) a mass‐specific metabolism that depends on mass in self‐replicators that are close to a lower size limit, and (4) a gradual unfolding of feed‐back selection by the density‐dependent interactive competition that follows from the population growth of self‐replication.

By integrating primary selection on mass‐specific metabolism into MR, I showed that the origin of replicating molecules starts a natural selection unfolding where the life‐history is selected as a balance between the energy that the average individual assimilates from the environment, and the energy that it uses on metabolism, mass, and reproduction. The primary driver of the selection is generally not the physiology, but the ecological feed‐back between the density–frequency‐dependent selection of interactive competition and the underlying population‐dynamic growth.

The Darwinian paradigm implies evolution by differential self‐replication, where the intrapopulation variant with the fastest self‐replication is selected over other variants. But the frequency‐independent selection of the physiology was found to select only for an initial transition from replicating molecules to self‐replicating cells with a minimum mass. A selection balance with individuals that are larger than prokaryotes, including larger unicells and multicellular animals with sexual reproduction, was found to be selected by the gradual population‐dynamic development of the density–frequency‐dependent selection of interactive competition. This mechanism implies that fast intrapopulation replication is maintained as the agent that selects. But the gradual development of the density–frequency‐dependence implies a shift, where the average rate of replication is selected first as a positive function of mass in virus and prokaryotes, then as a positive over invariant and finally declining function of mass in larger unicells, to a persistent decline with the selection of mass in multicellular animals.

Given suitable environmental conditions, the unfolding of the feed‐back is a unidirectional development that follows from a sustained selection for an increase in the net energy that is available for self‐replication. The evolution of major lifeforms is therefore found to occur as a deterministic succession from replicating molecules, over prokaryote and protozoa‐like self‐replicating cells, to the fully developed multicellular animal with sexual reproduction between females and males.

### 8.1 Species distributions

This evolution raises the question why a majority of organisms have remained at a unicellular level. The straightforward answer is that they have been unable, for some reason or the other, to be selected to an energetic level that can sustain the feed‐back selection for the multicellular animal.

If all phylogenetic lineages of mobile organisms were free to evolve by unconstrained selection across ecological resources, we would, at least in the long run, expect that they should evolve toward the multicellular animal with sexual reproduction. But interspecific interactions can exclude the individuals of smaller species from essential resources. Given a multitude of species, competitive exclusion will operate as a downward cascade that maintains a succession of smaller species that are constrained in their selection of net energy by the competitive interactions with similar but larger species. We may thus expect a distribution of species with net energetic states and selected body masses that range from a possible minimum to a maximum, with the maximum increasing over evolutionary time.

Such species distributions of body masses are maintained by a constant action and outbalancing of oppositely directed selection forces on net energy and mass. While the hypothesis that body mass distributions evolve by a neutral diffusion away from a lower size limit can produce a statistical description that appears adequate (e.g., Caluset & Erwin, 2008; Gould, 1988; Jablonski, 1997; McKinney, 1990; Shoemaker & Caluset, 2014; Stanley, 1973), it is, from a mechanistic point of view, most probably not only flawed, but also redundant as it is pointless to assume a neutral diffusion to explain an increase in size that is explained already by the selection of net energy and mass that is necessary for the very existence of organisms with non‐negligible body masses.

This selection predicts that there is no such thing as an optimal mass for a given niche, as traditionally assumed for body mass evolution (e.g., Bonner, 2006; Brown & Sibly, 2006; Gould, 1988; Schoener, 1969; Shoemaker & Caluset, 2014; Stanley, 1973). The body masses of multicellular animals are instead selected by intraspecific feed‐back selection, where the selection for a smaller mass by the quality–quantity trade‐off is outbalanced by the population‐dynamic feed‐back of interactive selection. This selection balance is scaled for the evolution of smaller or larger masses by the average net energy of the individuals in the population. Even if resource handling evolves to an optimum for a given set of resources, we expect no optimal mass because the net energy is given also by a persistent selection for an increase in metabolic pace.

It is essential to keep in mind that the interspecific interactions occur between individuals, and not directly between species or populations. The competitive exclusion from resources is therefore not necessarily the exclusion from an abundant and widespread resource that can maintain a large population, but more importantly the exclusion from a resource that is easy for the individuals to exploit for energy and other essential components. The resources that are exploited by the larger species are therefore not necessarily the resources that can sustain the largest biomass (Makarieva, Gorshkov, & Li, 2004; Trebilco, Baum, Salomon, & Dulvy, 2013). It is for similar resources that we expect the straight forward allometric relations where biomass ($nw\propto w^{1/2d}$) increases with mass and the energy ($nw\beta \propto w^{0}$) that is metabolized by the population is invariant of mass.

### 8.2 Theoretical background

MR is based on several concepts that were developed relatively independently of one another in the past. Some of these are (1) metabolism as a proxy for the rate at which organisms assimilate, transform and expend energy (e.g., Brown, Gillooly, Allen, & Savage, 2004; Calder, 1984; Humphries & McCann, 2014); (2) a biological time‐scale as the inverse of mass‐specific metabolism (e.g., Brody, 1945; Pearl, 1928); (3) that advanced metabolism is dependent upon a cell where the molecules of the metabolic pathways can concentrate (e.g., Maynard Smith & Szathmáry, 1995; Miller & Orgel, 1974; Oparin, 1957); (4) that natural selection is driven by the biochemical energetics of self‐replication (e.g., Brown, Marquet, & Taper, 1993; Lotka, 1922; Odum & Pinkerton, 1955; Van Valen, 1976); (5) that it is constrained by physiological trade‐offs and constraints (e.g. Charlesworth, 1980; Roff, 1992; Stearns, 1992), including a metabolism that depends on mass in self‐replicators with almost no mass (DeLong et al., 2010); (6) that it proceeds toward attractors like continuously stable strategies (e.g., Eshel & Motro, 1981; Maynard Smith & Price, 1973; Taylor, 1989); (7) that it is dependent upon the feed‐back ecology of density dependence (e.g., Anderson, 1971; Heino, Metz, & Kaitala, 1998; Rankin, 2007), including the density dependence of interactive competition (e.g., Abrams & Matsuda, 1994; Witting, 1997) that makes arms race models (e.g., Dawkins & Krebs, 1979; Maynard Smith & Brown, 1986; Parker, 1979) realistic; (8) that the unit of selection is the interacting unit that makes replication differential (Hull, 1980); (9) that higher‐level selection trade‐off fitness at the lower level for increased fitness at the higher level (Buss, 1987; Michod, 1999); (10) that the resulting short‐term evolution is contingent upon the current state of biology and the available mutations; and (11) that long‐term evolution is more like a deterministic path (Witting, 1997, 2008) that is laid down by the selection attractors that unfold from the origin of replicating molecules, including allometric exponents that evolve by the ecological geometry of optimal density regulation (Witting, 1995, 2017).

### 8.3 A sufficient Darwinian hypothesis

That an essential part of the long‐term evolution of life histories is deterministic from the origin of replicating molecules is in contrasts to the 20th Century paradigm, where evolution by natural selection, was seen as inherently contingent (e.g., Gould, 2002; Mayr, 1988; Maynard Smith & Szathmáry, 1995; Salthe, 1989; criticized by Conway‐Morris, 2003; Kauffman, 1993, 1995; Witting, 1997, 2008). In the strong interpretation, contingency implies an evolution that cannot be predicted a priori by natural selection, but can only be understood a posteriori from its historical development once it has actually occurred (Gould, 2002). This view underlies classical life‐history theory that developed contingent a posteriori selection models that explain traits from evolutionary assumptions on other traits in existing lifeforms (reviewed by Charlesworth, 1980; Roff, 1992; Stearns, 1992).

The contingent view is typically assuming populations that evolve toward an optimum for the niches that they occupy, with no inherent direction to natural selection per se (e.g., Bonner, 2006; Brown & Sibly, 2006; Caluset & Erwin, 2008; Gould, 1988; Shoemaker & Caluset, 2014; Stanley, 1973). An evolutionary size increase was seen as an adaptive advantage that may include an improved ability to capture prey, avoid predators, have greater reproductive success, decreased annual mortality, increased longevity, increased intelligence from increased brain size, expanded niche width, increased heat retention, etc. While these and many other factors may influence the evolution of mass by their influence on the population‐dynamic feed‐back, there is really no evidence to suggest that they are the essential factors that select mass (see Appendix 1). Most views of an adapting mass have insufficient mechanisms because they do not develop an explicit mathematical model for the natural selection of the mass that they are proposed to explain.

Explicit models of contingency explained large body masses (McLaren, 1966; Roff, 1981; Stearns & Koella, 1986), and allometric correlations (Kozlowski, Konarzewski, & Gawelczyk, 2003; Kozłowski & Weiner, 1997), from a reproductive rate that is positively correlated with mass across the body mass variation in a species. As reproduction is proportionally related to mass in many species (Peters,^1983^; Reiss, 1989), these models mimic natural selection on mass in those species. Yet, the approach is insufficient from a natural selection point of view because it does not explain the origin of the natural selection pressure on mass. The proportional dependence of reproduction on mass is instead explained by the deterministic model in this article, where it evolves as a consequence of the resource bias of interactive competition in populations with body masses that are selected to increase exponentially (Witting, 1997, 2003).

With Witting (2017) and this article, I aimed for a natural selection that is sufficient to explain the joint evolution of the metabolism, mass, allometries, and major transitions that define lifeforms from viruses over prokaryotes and larger unicells to multicellular animals. The proposed model can be seen as a sufficient Darwinian hypothesis that aims to explain the complete life‐history of the model organism from the origin of replicating molecules (Witting, 1997, 2008). It is not a model that attempts to explain the evolution of everything, but merely a model that is internally self‐consistent with the hypothesis that advanced lifeforms evolve as a deterministic consequence of the natural selection that unfolds from the origin of replicating molecules. This implies predictions from first principles of replication, without the estimation of parameters from data on evolved lifeforms.

### 8.4 The fallacy of observation

Contingent hypotheses are typically based on a frequency‐independent selection that allows for an easy interpretation of evolved interspecific relationships, as these are transferred more or less directly from the intraspecific correlations of fitness landscapes (see Figure 1a–c). Body mass allometries are no exception, and they provide clear examples that illustrate how the contingent approach of basing selection relations on observed relationships may lead to false conclusions of evolutionary causality.

The most obvious example is maybe the rate of replication that tends to decline to the negative 1/4 power of mass in multicellular animals (Fenchel, 1974). This indicates a strong frequency‐independent selection against mass, yet the very existence of the larger species shows that this apparent selection is flawed. The hint is that we should use this observed paradox to conclude that the natural selection of mass is at least frequency‐dependent, because frequency‐dependent selection is what we need in order to get evolution to proceed against a gradient of increased replication (see Appendix 1 for more details).

Take also Equation (6), where net energy is a product ϵ∝αβ between resource handling and mass‐specific metabolism. While this is supported by our logical reasoning of natural selection, it is not supported directly by the observed interspecific allometries. If we look across species in the major taxa of multicellular animals, we see a mass‐specific metabolism that declines with mass and net energy, suggesting that the dependence may not hold. And if we look at the macroevolutionary scale in Makarieva et al. (2005, 2008) and Kiørboe & Hirst (2014), we find that both gross and net energy increase sublinearly with mass, while the average mass‐specific metabolism tends to be independent of mass. This may indeed suggest that both net and gross energy are more dependent on mass than on mass‐specific metabolism, and this may easily be argued at least for unicells where gross energy uptake is likely to depend on the area of the cell.

It might therefore at first seem more intuitively correct to construct a model where mass is selected because gross and net energy are functionally dependent on mass. Yet, if possible at all, it is not a straight forward task to construct such a theory that is both internally consistent and consistent with intraspecific and interspecific observations (see Appendix 1). It is, for example, easily shown that a potential dependence of net energy on mass is generally not sufficiently strong to select for an increase in mass, not even in the case where gross and net energy are a direct function of the area of the cell.

While it may be tempting, as done above, to think in frequency‐independent selection and use the observed interspecific allometries to question basic functional relations like $\epsilon =\alpha \tilde{\beta }$, it is essential to keep in mind that this is a flawed approach. If observed interspecific allometries are to be compared with selection models, they should be compared with the interspecific predictions of the models and not with the basic relations that generate the intraspecific selection of the models.

As the natural selection of mass from a net energy that is given by handling and pace is based on density–frequency‐dependent feed‐back selection with overlaid time‐dilation from mass‐rescaling selection, there is no reason to expect that the intrinsic constraints that generate part of the selection should resemble the evolutionary relationships that are observed across the selected species. And this is also what we find, with a mass‐specific metabolism that, dependent on the evolutionary succession, is predicted to either increase or decline with mass despite of the positive dependence of gross and net energy on metabolic pace. This creates the pattern of Figure 4, where mass‐specific metabolism increases with mass in prokaryotes, where it is approximately independent of mass across protists and protozoa, where it declines with mass in taxa of multicellular animals, and where it is mass invariant on the macroevolutionary scale across all taxa. Hardly any of the theoretical allometries in Table 2 are predicted to be similar across these scales. The exceptions include gross and net energy that are predicted to scale sublinear ($\epsilon \propto w^{(2d-1)/2d}$) with mass at all scales, in agreement with observations by Kiørboe and Hirst (2014).

### 8.5 The primary selection of metabolism

My earlier work on sufficient population‐dynamic feed‐back selection predicted an exponential increase in net energy and mass (Witting, 1997, 2003), with a major transition between negligible‐sized low‐energy self‐replicators with asexual reproduction, and high‐energy organisms with large body masses, senescence, and sexual reproduction between a female and male individual, including additional transitions to cooperative breeding and eusocial colonies (Witting, 1997, 2002a, 2007, 2008). This work followed a widespread tradition where the interspecific variation in mass‐specific metabolism was seen as a consequence of a negative allometric scaling with mass (Witting, 1995).

In Witting (2017) and this article, I adjusted my first view on the natural selection of metabolism (Witting, 2003) and separated the resource assimilation parameter of my original model into resource handling and the pace of handling. This pace generates gross energy, and by defining net energy as the difference between gross energy and the total metabolism of the organism, Witting (2017) found the mass‐specific work of handling to be selected as mass‐specific metabolism. This implies primary selection for an increase in mass‐specific metabolism, an increase that generates part of the net energy that is a precondition for the selection of mass, and the associated secondary rescaling of mass‐specific metabolism with the evolutionary changes in mass.

In the earlier version of the theory, small‐bodied asexual self‐replicators were predicted to be a single group with no explicit allometries. But by integrating primary selection on metabolism into the theory, the predicted asexual group unfolded into three subgroups with life‐histories and allometries that resemble those of respectively viruses, prokaryotes, and larger unicells. The primary selection difference between these taxa is reflected in the dependence of mass‐specific metabolism on the minimum mass that is required to sustain metabolism, with transitions between the three lifeforms reflecting transitions in this dependence.

### 8.6 The metabolizing cell

The dependence of mass‐specific metabolism on mass in small replicators should be seen in relation to the evolution of the cell. Extremely low levels of metabolism are not in principle dependent upon the development of a compartment like a cell. But it is difficult to imagine the evolution of an advanced intrinsic metabolism unless it is connected with the formation of a cell where the metabolic molecules can concentrate (e.g., Koch & Silver, 2005; Maynard Smith & Szathmáry, 1995; Michod, 1999; Miller & Orgel, 1974; Oparin, 1957; Wächtershäuser, 2000). Taking this point of view, I found that the increase in net energy for self‐replication with increased metabolism is the primary selection that drives the evolution of the cell and all its associated mass. This implies that a self‐replicating cell with heredity and an internal metabolism can be selected only from the subsets of the potential biochemistry that have a maximum exponent for the primary dependence of mass‐specific metabolism on mass that is larger than unity (${\hat{\beta }}_{\beta ,0}>1$).

This result agrees with the evolutionary emergence of the cell, as well as the evolutionary transition between unicellular self‐replicators and multicellular organisms. With the cell being the compartment that organizes metabolic pathways, it follows that the quality–quantity trade‐off is selecting for virus‐like replicators with no metabolism and the absence of a cell when ${\hat{\beta }}_{\beta ,0}<1$. Unicellular self‐replicators may evolve when ${\hat{\beta }}_{\beta ,0}>1$, and as these are selected to have the minimum possible mass that is required for their mass‐specific metabolism, they are, as a consequence of this, selected to have no more than a single cell only. High‐energy organisms with a sexual reproduction that is selected by interactive competition are instead predicted to have a mass that is larger than the required minimum for metabolism. Hence, they are free to evolve multicellularity with specialized and co‐operating cells that enhance the behavioral performance of the individual and allow for the evolution of more efficient transportation networks within the organism.

DeLong et al. (2010) propose in comparison that (1) the rapid increase in metabolic rate with increasing cell size in prokaryotes reflects an increase in the number of genes, (2) that the approximately linear scaling in protists reflects a linear increase in the ATP synthesis of organelles, and (3) that the sublinear increase in metazoans reflects physiological constraints on resource distribution through vascular systems (West, Brown, & Enquist, 1997). But when we build an explicit model on the natural selection of metabolism and mass, as done in the this article, we find that the evolutionary causality of this reasoning is premature.

The natural selection suggests instead that it is not an increase in genome size that drives the evolution of a larger cell with a stronger metabolism; the larger genome is only a secondary effect that is selected because a larger heritable code is a necessity to code for a metabolism that is selected, by the increase in the net energy of the replicator, to increase super‐linearly with mass. It is also not an increase in the ATP synthesis of organelles that dives the evolution of larger protists, but instead a metabolism that is selected to increase about linearly with mass that selects for a linear increase in the ATP synthesis of organelles. And the vascular system of multicellular animals is optimized for a metabolic pace that is selected to decline to the −1/2d power of mass by the mass‐rescaling selection of the life‐history.

The increase in genome size with mass in prokaryotes, the increase in organelles with mass in protists, and the evolution of vascular transportation systems in metazoan do all reflect genotypic and phenotypic solutions that are necessary for the organism to evolve along the path that is laid down by the natural selection that unfolds from the origin of replicating molecules.

## 9 CONCLUSION

The proposed natural selection is so far unique in the sense that it is the only one that is sufficient to explain the joint evolution of metabolism, mass, major life‐history transitions, and allometric exponents from viruses to multicellular animals. These transitions and exponents are explained almost as parsimoniously as possible as they evolve, not from separate processes, but from the primary selection on metabolism and mass itself; the selection that is necessary for the very existence of the organisms that are a precondition for the existence of life‐history transitions and allometries.

These organisms have to balance their energy between metabolism, mass, and self‐replication. The observed balance is only one of infinitely many potential possibilities, and the selection that unfolds from replicating molecules was found in this article to explain the overall balance from viruses over prokaryotes and larger unicells to multicellular animals.

### 9.1 Eleven conclusions

1. Mass‐specific metabolism is selected as the pace of the resource handling that generates net energy for self‐replication, with persistent selection for a continued increase in mass‐specific metabolism and net energy.
2. The quality–quantity trade‐off, where parents can produce many small or a few large offspring from the same amount of energy, is constantly selecting for the near absence of mass. This implies that the intraspecific dependence of net energy on mass needs to increase at least linearly with mass in order to outbalance the quality–quantity trade‐off and select for larger body masses.
3. A selection increase in mass generates a mass‐rescaling, where life‐history and ecological traits evolve in response to the evolutionary changes in mass. This mass‐rescaling selection is initiated by a metabolic trade‐off that selects for a decline in metabolism and a dilation of the reproductive period with an increase in mass. This prevents a decline in net energy and fitness on the per‐generation time‐scale of natural selection.
4. The physiological mass‐rescaling of the life‐history imposes mass‐rescaling on the interactive foraging behavior that determines the selection optimum of density regulation. This ecological optimum explains the 1/4 exponents of Kleiber scaling as the two‐dimensional case of the more general 1/2*d*, where *d* is the spatial dimensionality of the intraspecific interactive behavior.
5. Body mass allometries depend also on a metabolic‐rescaling, where the life‐history is rescaled by the relative importance of mass‐specific metabolism for the evolution of mass. The −1/2d exponent for mass‐specific metabolism evolves when the interspecific body mass variation is invariant with respect to the primary selection of mass‐specific metabolism, and the corresponding exponent is (2d−1)/2d when all of the variation in mass is evolving from primary selection on mass‐specific metabolism.
6. Small self‐replicators have a mass‐specific metabolism that is dependent on the mass of the molecules in metabolic pathways, and on the mass of the cell where the metabolic molecules concentrate, including the mass of the heritable code for the cell and the metabolic pathways.
7. Replicating molecules with no metabolism, no cell, and practically no mass—like viruses—are predicted to be selected from lineages where an initial mass‐specific metabolism is increasing sublinearly with mass.
8. Small single‐celled self‐replicators with an internal metabolism are selected from lineages where the initial mass‐specific metabolism is increasing stronger than linearly with mass. Given the absence of an intraspecific resource bias from interactive competition, these self‐replicating cells are predicted to have asexual reproduction and a mass‐specific metabolism that increases to the 5/6 power of mass (given 3D ecology), as empirically estimated in prokaryotes.
9. Larger single‐celled self‐replicators with a more developed metabolism and asexual reproduction are selected from a population‐dynamic feed‐back of interactive competition that is unfolding gradually from an evolutionary increase in net energy. These interacting self‐replicators have an allometric exponent for mass‐specific metabolism that declines from 5/6 over zero to −1/6 with an increasing mass (given 3D ecology), as empirically indicated for protozoa.
10. Multicellular animals with large body masses and sexual reproduction are predicted to be selected from the intraspecific interactive competition in high‐energy lineages with a fully developed population‐dynamic feed‐back selection. Given a species distribution that diversity across ecological niches, the predicted interspecific exponent for mass‐specific metabolism is −1/2d, with −1/4↔−1/6 transitions being observed quite commonly between terrestrial and pelagic taxa.
11. Given a mass‐specific metabolism that evolves around an upper bound, mass‐specific metabolism is predicted to be body mass invariant on the macroevolutionary scale from prokaryotes to mammals, as empirically estimated by the absence of an allometric correlation.

## CONFLICT OF INTEREST

None declared.

## APPENDIX 1

### Physiological selection on mass

This appendix discusses some general considerations and definitions in relation to the frequency‐independent selection that the physiology is imposing on mass. Apart from a dependence of mass‐specific metabolism on mass in self‐replicators with minimum body masses (see Section 3), I treat the life‐history as being functionally independent of mass.

This assumption was found to work in relation to the long‐term evolution of mass. But there may be cases where life‐history components are functionally dependent on mass. Larger individuals may, for example, be less likely to be taken by predators. This may influence the distribution of evolved body masses, but it will generally not explain the evolution of large masses. The predation hypothesis does not apply to top‐predators, and to explain the evolution of mass it requires that the survival parameter *p* is increasing at least proportional with mass, which is implausible at least in general.

Another apparent link is net energy ($\epsilon =\alpha \tilde{\beta }$) that nearly always is strongly positively correlated with mass. There seems, however, to be no general mechanism that will explain the evolution of large body masses from a resource assimilation that is larger in larger organisms because resource assimilation is functionally dependent on mass. So I treat resource assimilation as functionally independent of mass, aiming to show that large masses evolve by natural selection in high‐energy species (high‐energy organisms could instead have evolved small masses and very high replication rates).

For this, I assume that it is generally not a functional dependence of α on *w* that is the cause for the evolution of large body masses. Nevertheless, the handling of resources may indeed increase mechanistically with mass. But this dependency is assumed to be so small that it will generally not affect our evolutionary predictions if it is ignored. To show that large species can evolve by natural selection, we need to predict that they evolve a strong positive correlation between *w* and α (i.e., if $\beta_{\beta }$ is constant); and with α being functionally independent of *w*, we predict the evolution of a mass that often increases proportional with α (Witting, 2017). This is a stronger link than we can expect for almost any functional dependence of α on *w*.

Maybe the strongest functional dependence of α on *w* is in organisms that obtain resources through their complete surface. In this case we may expect $\alpha \propto w^{2/3}$ from the relationship between the surface area and the volume (mass) of an individual. Yet, from Equations (1) and (6) we find that α must increase at least proportional with mass for a large body mass to evolve as a response to a positive dependence of α on *w*. Hence, even in this special case with a very strong dependence, we cannot straightforwardly explain large masses as a selection consequence of a positive linking of α to *w*.

The availability of resources ρ, which is included in the α parameter (as $\alpha =\overset{`}{\alpha }\rho$, where $\overset{`}{\alpha }$ is intrinsic handling), might also increase with mass if an increased mass allows the organisms to exploit a larger spectrum of resources. While such a positive relationship may exist in some cases, and while this may influence the distribution of evolved body masses, it is unlikely a general principle that will explain the evolution of large masses. Quite generally, I treat resource availability as a parameter that is not directly part of the phenotype and thus not affected directly by the natural selection of my model (but it may be affected indirectly by the evolution of the phenotype). Nevertheless, variation in $\rho^{**}$ is affecting the realized net energy, and this variation is selected into variation in mass; as part of the variation in α that is selected into variation in mass.

As none of these potential links of the life‐history to mass will explain a general selection for large body masses, I will usually treat the life‐history as being independent of mass except for the dependence that evolves from the natural selection of my equations. The selection gradient on log body mass

(A1) ∂r/∂lnw=−1,

that is imposed by the physiology is thus generally around minus one from Equation (1). This reflects a replication cost of mass that is selecting for a minimum mass

(A2) $$w\to {\underline{w}}^{*\underline{*}}.$$

I treat this downward pull as a fundamental background selection that any natural selection for mass needs to outbalance in order to push evolution beyond the replicating molecules at the origin of life.

### A1 Classical selection theory

The downward pull of Equation A1 is generally neglected in the contingent selection of the classical life‐history theory (McLaren, 1966; Roff, 1981, 1986; Stearns & Crandall, 1981; Stearns & Koella, 1986). The contingency makes it possible to assume an intraspecific relation between mass and replication that is based on observations instead of energetic trade‐offs. The reproductive rate was thus assumed to be about proportional to mass, as observed in many populations (Peters, 1983; Reiss, 1989) and supported by widespread natural selection for an increased mass in natural species (Kingsolver & Pfennig, 2004).

To describe this by a simple contingent model, we note from Equation (1) that intraspecific proportionality between reproduction and mass requires net energy to increase to the second power of mass ($\epsilon \propto w^{2}$). Then, if survival is negatively related to mass (e.g., as $p\propto e^{-cw}$), we may obtain a fitness profile (landscape) $r_{i}=\ln w_{i}-cw_{i}$ that describes fitness as a function of the body mass variation ($w_{i}$) in the population (Figure 1a). The differential of the profile ($\partial r_{i}/\partial w_{i|w_{i}=w}=1/w-c$) at the limit of the average variant (*w*) is the selection gradient (Figure 1b) that describes selection as a function of the average body mass. And the evolutionary equilibrium ($w^{**}=1/c$) is the optimum of the selection integral [$\int \left(\partial r_{i}/\partial w_{i|w_{i}=w}\right)dw$; Figure 1c] that integrates the selection gradient across the potential evolution in the average mass of the population.

The more recent approach of metabolic ecology (Brown & Sibly, 2006) assumes, somewhat contrary to evidence, that the variation in the intraspecific birth rate ($b_{i}\propto w_{i}^{-1/4}$) follows the interspecific allometry for biotic periods, with an intraspecific birth rate that declines with mass instead of increasing as often observed. Large species may then evolve by a survival rate that increases with mass. While metabolic ecology has changed the selection roles of reproduction and survival relative to traditional life‐history theory, the two approaches are structurally identical as they are based on constant relative fitnesses with frequency‐independent selection. The outline in Figure 1a–c is therefore applicable to both, as well as to other arguments and models with frequency‐independent fitness on mass (e.g., Charlesworth, 1994; Charnov, 2011; DeLong, 2012; Schoener, 1969; Stanley, 1973).

The plots in Figure 1a–c show a direct relationship between the fitness landscape (profile) and the potential evolution along the selection integral, with the most fit variant in any population being the average variant at the evolutionary equilibrium. This relation reflects evolution by Fisher's (1930) fundamental theorem that is selecting for an increase in what is known as the average fitness of the population; measured either by the intrinsic growth rate (*r*) and/or by the carrying capacity (*n**) of the population (Charlesworth, 1971; Roughgarden, 1971; Witting, 2000).

One inherent problem with these approaches is the constant relative fitnesses that make the intrinsic components of fitness and of fitness‐related traits like reproduction and survival, scale identically within and across species (Witting, 2000, 2002b), as illustrated by the similar curves in Figure 1a,c. This restriction is quite generally contradicted by data in at least birds and mammals, where lifetime reproduction and survival tend to be independent of mass across species (Witting, 1995).

For interspecific allometries in multicellular species, *r* tends to scale to the negative 1/4 power of mass (Fenchel, 1974) and *n** to the negative 3/4 power (Damuth, 1981). In combination with a life‐history theory that is based on constant relative fitnesses, these data imply a decline in fitness (*r* or *n**) with mass, and this generates the paradox of constant selection for the near absence of mass in basically all natural species. Realized reproduction and survival could, of course, scale differently at the two scales if resource exploitation was a function of body mass across species. But the resource consumptions of populations are often independent of mass across natural species (Calder, 1984; Damuth, 1981; Peters, 1983).

We may conclude that the combination of contingency and frequency‐independent selection is insufficient to explain the joint evolution of mass and allometries.
